# Supplementary figures and images for: kSanity: A k-mer based application for precision bacterial strain detection and quantification
Source: bioRxiv. 2025 Sep 9:2025.09.04.674052. Preprint. [Version 1] doi: 10.1101/2025.09.04.674052 (PMC12439994; doi:10.1101/2025.09.04.674052)

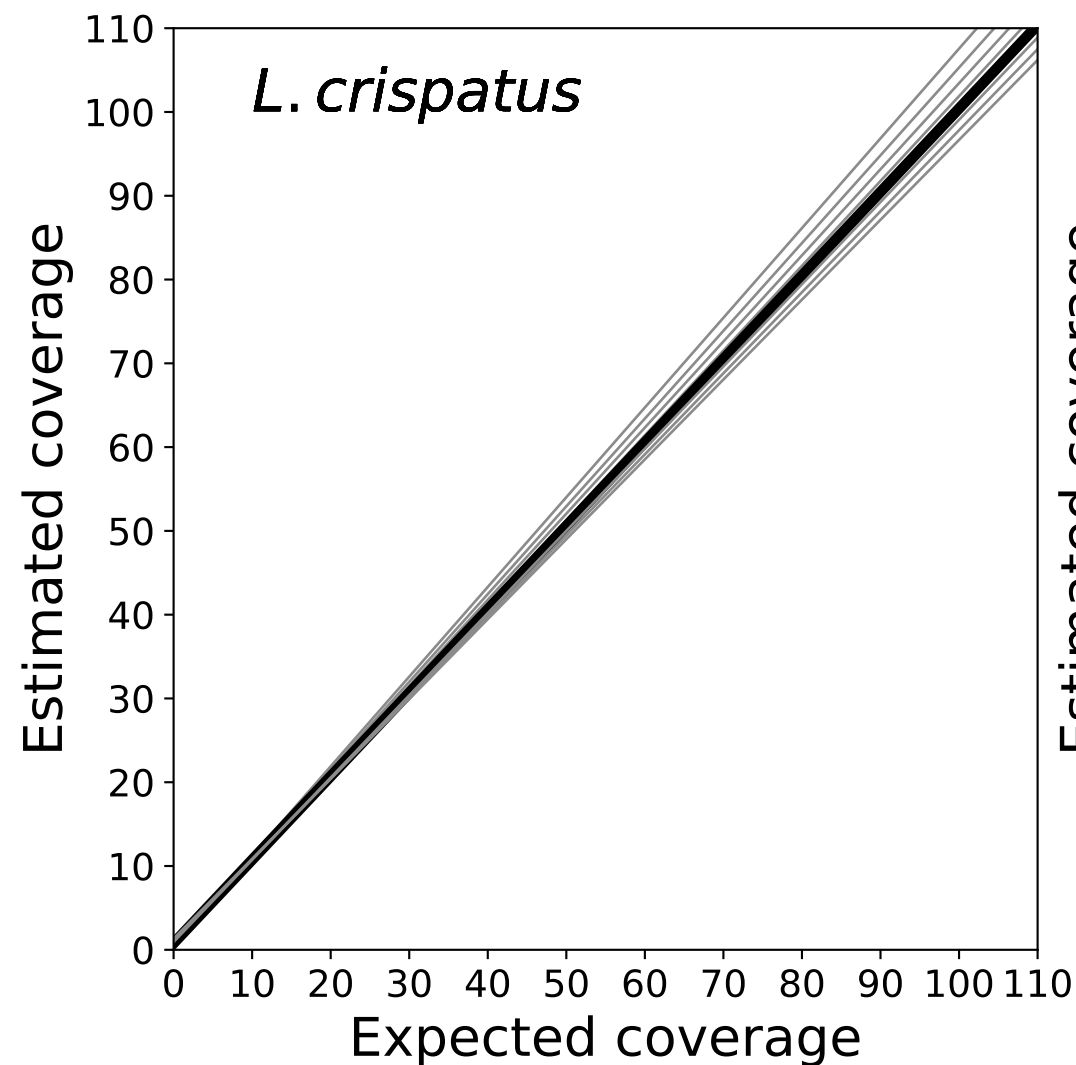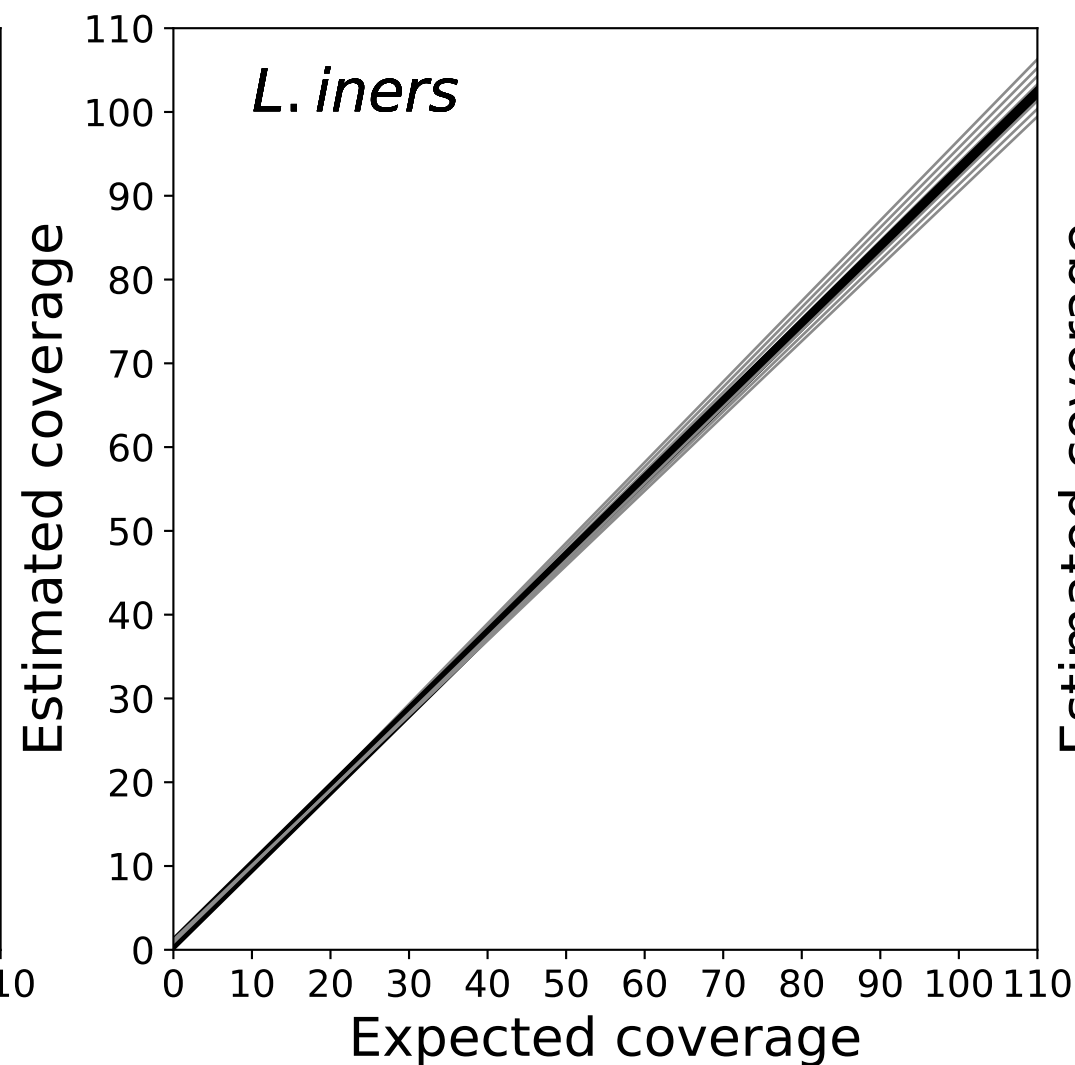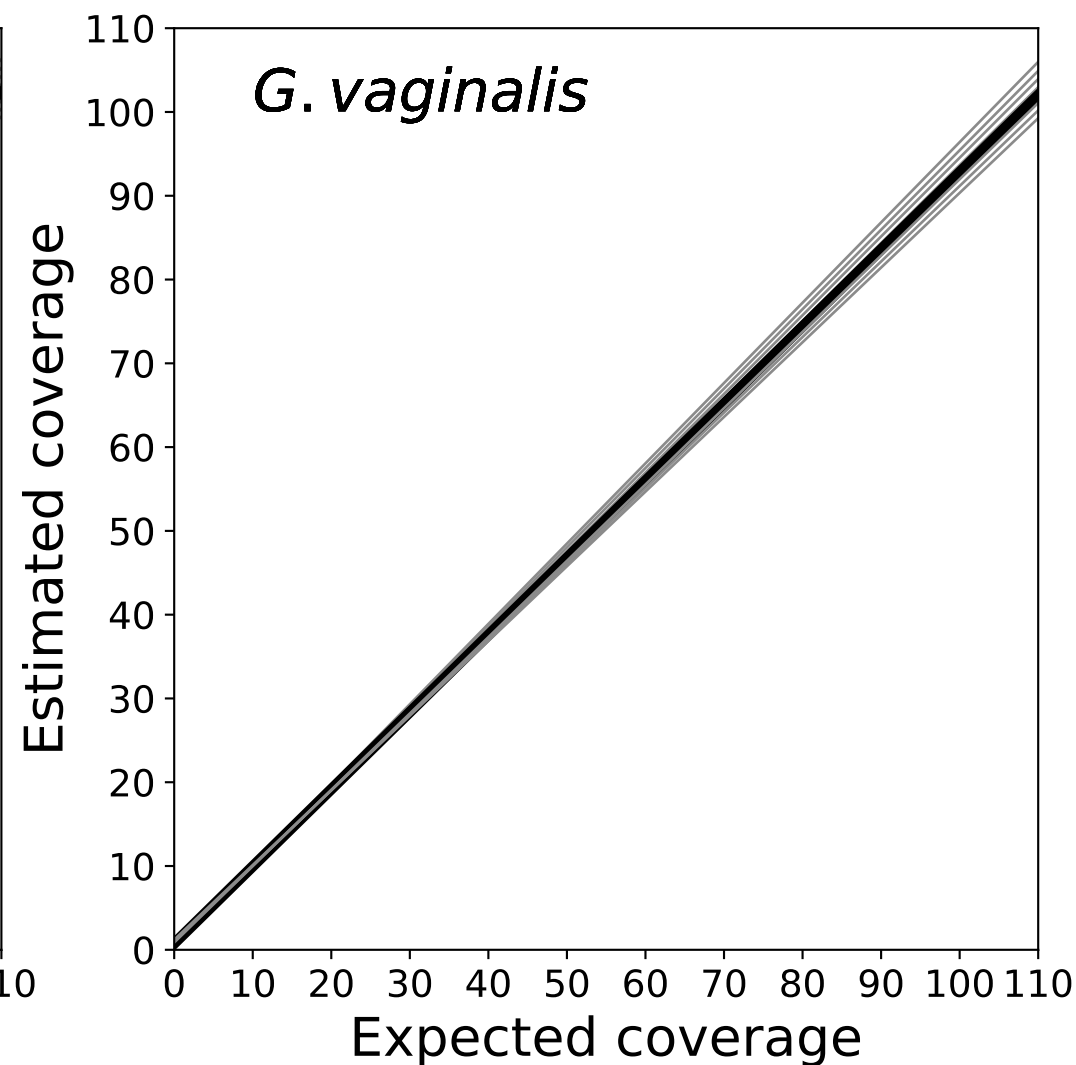

Supplement: Supplement 1 — Supplementary Figure 1: Linear regression lines relating the expected and estimated coverages for different values of k. The bold line represents the value of k used in all of the presented analyses (k=55). [file media-1.pdf]
